# Programmed cell death-1 (PD-1) at the heart of heterologous prime-boost vaccines and regulation of CD8^+ ^T cell immunity

**DOI:** 10.1186/1479-5876-8-132

**Published:** 2010-12-14

**Authors:** Adrian Bot, Zhiyong Qiu, Raymond Wong, Mihail Obrocea, Kent A Smith

**Affiliations:** 1 MannKind Corporation, 28903 North Avenue Paine, Valencia, CA 91355. USA

## Abstract

Developing new vaccination strategies and optimizing current vaccines through heterologous prime-boost carries the promise of integrating the benefits of different yet synergistic vectors. It has been widely thought that the increased immunity afforded by heterologous prime-boost vaccination is mainly due to the minimization of immune responses to the carrier vectors, which allows a progressive build up of immunity against defined epitopes and the subsequent induction of broader immune responses against pathogens. Focusing on CD8^+ ^T cells, we put forward a different yet complementary hypothesis based primarily on the systematic analysis of DNA vaccines as priming agents. This hypothesis relies on the finding that during the initiation of immune response, acquisition of co-inhibitory receptors such as programmed cell death-1 (PD-1) is determined by the pattern of antigen exposure in conjunction with Toll-like receptor (TLR)-dependent stimulation, critically affecting the magnitude and profile of secondary immunity. This hypothesis, based upon the acquisition and co-regulation of pivotal inhibitory receptors by CD8^+ ^T cells, offers a rationale for gene-based immunization as an effective priming strategy and, in addition, outlines a new dimension to immune homeostasis during immune reaction to pathogens. Finally, this model implies that new and optimized immunization approaches for cancer and certain viral infections must induce highly efficacious T cells, refractory to a broad range of immune-inhibiting mechanisms, rather than solely or primarily focusing on the generation of large pools of vaccine-specific lymphocytes.

## The 'magic' of heterologous prime-boost vaccination

Vaccines are arguably the best medical tools we have at our disposal to fight widespread infectious diseases. Despite decades of vaccine research and development against life-threatening infectious diseases with global impact [1], culminating with the recent licensing of vaccines against human papillomaviruses (HPV) [2], a key cause of cervical cancer, successes have been confined primarily to prophylaxis. Vaccination has also been extensively researched for the prevention of HIV infection. Therapeutic immunization for cancer or chronic viral infection, however, brings in a new set of lessons and challenges with a few successes to date, such as treatment of HPV-related lesions [3]. It became rapidly evident that the conventional paradigm of eliciting, amplifying, and maintaining immune responses with conventional vectors and homologous prime-boost approaches fell short of expectations in the clinic due to suboptimal immune response results. Two decades since the first cloning of tumor antigens [4], multiple vaccines are currently in development. Thus far, however, sipuleucel T (Provenge^®^) is the only approved therapeutic cancer vaccine in the US to date, consisting of autologous DCs expressing prostate acid phosphatase (PAP) and producing granulocyte macrophage colony-stimulating factor (GM-CSF) to treat hormone-refractory prostate cancer [5].

The HIV vaccine field has unquestionably been at the forefront of vaccine research, exploring potent immunization strategies comprised of synthetic vectors rather than cell-based vaccines. This is in contrast to efforts in cancer vaccine development where cell-based vaccines currently lead the field, while many synthetic and viral vector approaches are in clinical development [6,7]. Nevertheless, homologous prime-boost approaches for the prophylaxis of HIV, such as the Vaxgene program, showed no significant protective effects in man [8]. While in parallel, emerging evidence over the last two decades showed that novel prime-boost protocols integrating different vectors such as recombinant viruses and proteins [9,10] did yield considerably higher immune responses with protective capability in several animal models. With the advent of other vectors such as DNA vaccines, and a range of recombinant microbial vectors including alpha virus replicons, research in the area of heterologous prime-boost vaccination against HIV has expanded and resulted in hundreds of preclinical and clinical studies. Interestingly, the most promising clinical regimens to date include: i) the RV144 landmark HIV 'Thai trial' which utilized recombinant viral priming followed by a protein boost and was the first to show modest yet statistically significant evidence of HIV vaccine efficacy in man [10]; ii) DNA priming coupled with protein [11]; or iii) DNA priming followed by a recombinant virus boost [12].

Significant evidence points to two major reasons why heterologous prime-boost vaccination is a more promising strategy compared to homologous prime-boosting: i) diminished anti-vector antibody responses [13] known to interfere with immunity against target epitopes through the clearance and degradation of vaccine via vaccine-antibody immune complexes; and ii) there is the potential for different vectors to work synergistically by inducing complementary arms of the immune response to jointly control complex pathogenic processes and overcome immune escape mechanisms. For example, while recombinant proteins are quite effective at inducing B and Th immunity, viral vectors can be more effective at inducing cytotoxic T cells [14].

**Figure 1:**
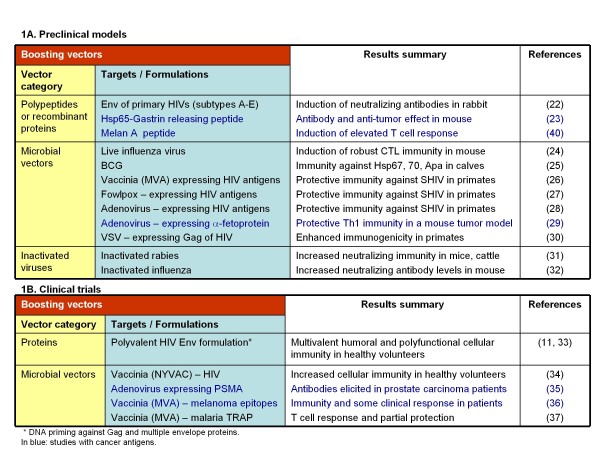
**Representative studies to date, evaluating DNA priming - heterologous boosting**.

DNA vaccine vectors offer several advantages, including the potential to elicit MHC class I-restricted immunity, reduced induction of anti-vector antibody responses, and reliance on a simple manufacturing process [15]. Nevertheless, DNA vaccination alone has yielded disappointing results in numerous clinical trials due to modest immune responses [16]. These results were largely attributed to low levels of vector-encoded antigen, resulting in low numbers of APCs expressing target epitopes, and subsequent inferior T cell stimulation and expansion *in vivo *[17]. Furthermore, intra-dermal gene-gun delivery [18], intra-lymphatic administration [19,20], or other enhancing approaches such as electroporation [21], have only partially improved the immune response achievable by DNA vaccination alone. Nevertheless, the potential of immune priming without the generation of interfering anti-vector antibodies has positioned DNA vaccines (Figure 1) as a primary component of several heterologous prime-boost vaccines in development for the treatment of diseases such as HIV, other microbes and cancer [11,22-37]. In addition, such protocols offer a more practical alternative for active immunotherapy of cancer and other diseases since they rely on synthetic or 'off the shelf' vectors, as compared to personalized DC-based vaccines [38].

The optimal positioning of current and future DNA vectors within innovative heterologous prime-boost immunization regimens requires a deeper understanding of the mechanism of action of DNA vaccination. A key observation from many studies to date is that interchanging the order of vectors utilized in these regimens has a dramatic impact on the resulting immune response. For example, while DNA priming followed by a virus boost resulted in significant epitope-specific responses, viral priming followed by DNA boost failed to reproduce this level of specific immunity [39]. A similar result was observed with other vectors in a distinct model, clearly supporting a precise sequence of administration of vectors as a major factor determining the magnitude of immunity [40], although this hypothesis still requires further testing in other heterologous prime-boost vaccine protocols. This asymmetry between priming and boosting vectors could very well be at the heart of both the mechanism and advantage of heterologous prime-boost regimens. Therefore, the remainder of this review will focus on this key feature and its underlying mechanism, with emphasis on DNA vaccines as priming agents and CD8^+ ^T cell immunity as the desired outcome, as it pertains to the control of cancer and chronic viral infections. Moreover, although we focus on the functionality of CD8^+ ^T cells in this review, we recognize the importance of CD4^+ ^T cells and the possibility that these cells may influence the outcome of vaccine protocols with respect to PD-1 expression by CD8^+ ^T cells.

## PD-1 and co-inhibitory receptors: a new dimension to prime-boosting and immune regulation

The fundamental concept behind heterologous prime-boost vaccination is the synergistic contribution of two categories of vectors to induce enhanced immunity against given epitopes. To investigate the immune mechanisms underlying this process, we initiated a systematic evaluation utilizing a reductionist approach that encompasses simple vectors with well-defined MHC class I-restricted epitopes. Using a Melan A/MART-1 preclinical experimental model, we developed a strategy that greatly enhances the immune properties of non-replicating vectors and biological response modifiers by direct intra-nodal administration of plasmid and peptide [19,41]. We showed that the sequence and the route of administration of plasmid and peptide were absolutely essential to achieve improved antigen-specific CD8^+ ^T cell immune responses [40]. While intra-lymph node priming with DNA (plasmid) and boosting with peptide afforded a robust expansion of epitope-specific CD8^+ ^T cells (on the order of 1/2 - 1/10 specific T cells/total CD8^+ ^T cells), reversing the order of the vectors resulted in a limited overall T cell expansion (~1/100 - 1/1000 or less, of specific T cells/total CD8^+ ^T cells) within the same range of homologous prime-boost vaccination [40]. A closer look at the immunity primed by plasmid showed that, in stark contrast to peptide priming, the epitope-specific CD8^+ ^T cells, although few in numbers (~1/100 specific/total CD8^+ ^T cells), had some strikingly distinguishing features. Within the population of CD8^+ ^T cells initiated by plasmid, we found a significant frequency of the lymphatic migration marker CD62L^+ ^(central/lymphoid-memory) epitope-specific CD8^+ ^T cells with a limited capability to produce proinflammatory cytokines upon peptide stimulation *ex vivo*. Nevertheless, these DNA vaccine-primed cells showed long-term persistence *in vivo *and displayed a high expansion potential following *in vivo *or *in vitro *re-exposure to antigen, associated with a rapid loss of CD62L and a broadening of their functional capabilities [40].

**Figure 2:**
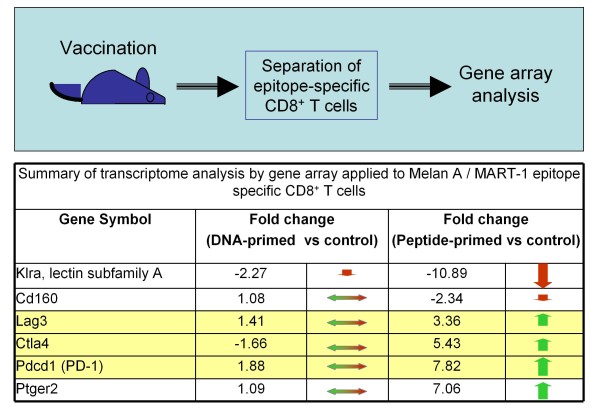
**Differential co-expression of inhibitory receptors by CD8^+ ^T cells depending on priming**. In brief, epitope-specific T cells from immunized mice were highly purified and analyzed without additional stimulation. Gene expression patterns were defined using hierarchical clustering; CD8^+ ^T cells from naïve mice were used as a reference control. The bottom half of the figure summarizes the results pertaining to expression of inhibitory receptors such as PD-1, as average fold change of gene expression relative to control. There was coordinated up-regulation of gene expression corresponding to membrane receptors with inhibitory activity (yellow shaded section: Lag3, CTLA-4 and PD-1) in CD8^+ ^T cells primed by peptide without adjuvant, but not DNA vaccine (summary of results in ref. [42]).

This obviously raised the question: Does priming with a DNA vaccine result in CD8^+ ^T cells that are more resilient to negative regulatory mechanisms that would otherwise impose restrictions on the expansion and activity of this key subset of T cells? To test our hypothesis, we compared the global gene expression in epitope-specific CD8^+ ^T cells generated by vaccination against Melan A/MART-1 with plasmid versus peptide in mouse [42]. We found numerous differences in regards to the transcriptome, most notably at the level of expression of genes encoding inhibitory receptors (Figure 2). More specifically, PD-1, CTLA-4, Lag-3 and the prostaglandin receptor Ptger2 were all significantly up-regulated in antigen-specific CD8^+ ^T cells from peptide (but not DNA) immunized mice, with the latter retaining a more 'naïve-like' phenotype from this point of view. In contrast, a member of the Klr family controlling the natural killer activity of lymphocytes was vastly down-regulated in CD8^+ ^T cells primed with peptide. Previous evidence also suggested that DNA vaccination elicited specific T cells with low PD-1 expression levels [43,44].

**Figure 3:**
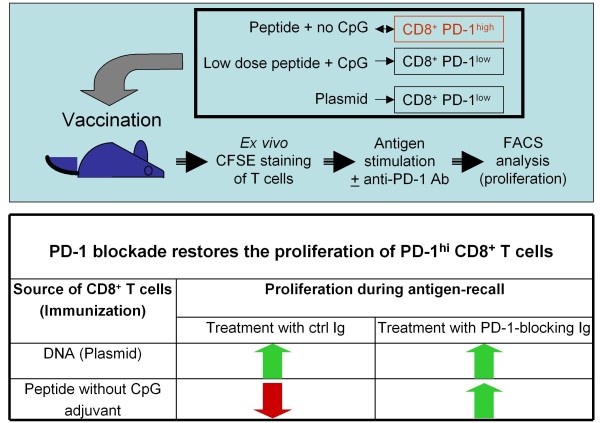
**The responsiveness of CD8^+ ^T cells is "imprinted" during the priming phase through PD-1 acquisition**. The upper panel depicts the general methodology: mice were immunized by various regimens and specific T cells were restimulated ex vivo with HLA-A*0201-binding human Melan A 26-35 native peptide (EAAGIGILTV), in the presence of PD-1 blocking antibodies or control immunoglobulin. Ex vivo T cell proliferation was measured using a standard CFSE staining assay. The bottom panel depicts a summary of the results comparing the essential groups: T cells from Melan A plasmid versus Melan A 26-35 analogue peptide (ELAGIGILTV) immunized mice. While the epitope-specific T cells from DNA vaccinated mice had low PD-1 expression and high proliferative potential persistently, the T cells from peptide immunized mice had high PD-1 expression and low proliferative potential; however, their proliferation could be easily restored through blocking PD-1/PD-1L interaction, speaking to the critical role of PD-1 in determining the fate of CD8^+ ^T cells post-priming (summary of results in refs. [42] and [48]).

This tandem co-regulation of inhibitory receptors [45-47] raised the possibility that this phenomenon, consisting of the generation of specific T cells that fail to up-regulate PD-1, extends beyond DNA vaccination. We investigated this concept by utilizing the opportunity afforded by intra-lymph node administration to evaluate the immune profile of peptide epitopes and biological response modifiers in their simplest form. Intriguingly, a rather low dose of peptide co-administered with robust doses of CpG (TLR9 ligand) resulted in Melan A/MART-1-specific CD8^+ ^T cells with low PD-1 expression levels [48], reproducing essentially the profile achieved by DNA vaccination (Figure 2). In stark contrast, a peptide dose increase or CpG dose reduction yielded increased levels of PD-1 expression on specific CD8^+ ^T cells. The induction of T cells with a high PD-1 expression level by peptide immunization alone may be due to co-presentation by professional and non-professional APCs alike. Co-administration of TLR ligands (such as CpG motifs and others) are expected to activate of APCs resulting in a favorable PD-1 profile [49-51]. As far as we know, the molecular mechanisms for these findings remain to be elucidated. Complementing these results, *ex vivo *antigen restimulation with simultaneous anti-PD-1 blockade restored the proliferation of PD-1^high ^CD8^+ ^T cells isolated from mice immunized with peptide only to levels similar to that of T cells from mice immunized with peptide + CpG or plasmid alone (Figure 3). This result strongly supports the functional relevance of this co-inhibitory molecule as a major regulator of CD8^+ ^T cell activity in the context of DNA priming- heterologous boosting and beyond. Furthermore, this nicely complements previous observations obtained with OVA-specific CD8^+ ^T cells defective in PD-1 expression in an autoimmune setting, showing the pivotal negative regulatory role of PD-1 both at the level of T cell expansion as well as during *in situ *activity [52]. In this experimental setting, PD-1^-/- ^OVA-specific T cells were adoptively transferred into transgenic mice expressing the antigen under the rat insulin promoter. The PD-1^-/- ^T cells proliferated to a higher extent in draining lymph nodes and caused insulitis and diabetes, in dramatic contrast to wild-type PD-1-competent T cells which were unable to mediate a similar outcome.

**Figure 4:**
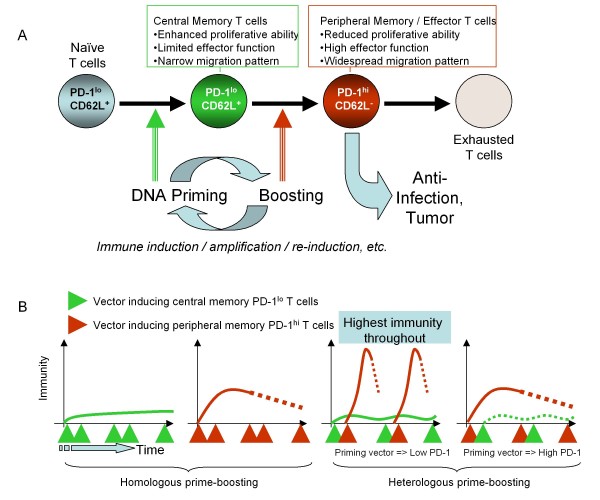
**The mechanism of prime-boosting in relation to PD-1-expression and central memory T cells**. The flowchart in Figure 4A depicts schematically a proposed mechanism explaining the effectiveness of DNA priming - heterologous boosting in achieving superior immunity in immune competent organisms. Alternating DNA priming with heterologous boosting (viral vectors, recombinant proteins, peptides, cells, or cell lysates), achieves alternating production of 'central-memory' low PD-1 cells and highly differentiated effector T cells, respectively. Figure 4B is a temporal perspective on the synergy and differential output of priming and boosting vectors/regimens, respectively. It offers an explanation to why the exact prime-boost sequence is important based on the differential capability of vectors or regimens to elicit T cells with different properties such as susceptibility to negative regulatory mechanisms.

**Figure 5:**
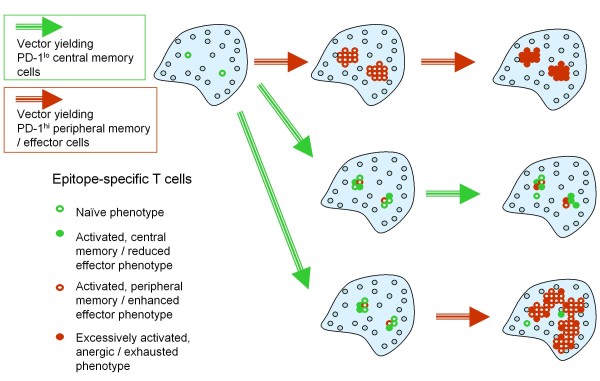
**Schematic representation of the kinetics of various subsets of T cells within secondary lymphoid organs**. This is a complementary perspective to that in Figure 4B, providing a rationale to why a specific sequence of priming and boosting is important to generating an elevated immune response.

With regard to the basic mechanisms of DNA priming/heterologous boosting, the following model thus emerges (Figure 4). Effective priming agents such as DNA vaccines induce a population of antigen-specific T cells with a central-memory phenotype (CD62L^+^) that reside within lymphoid organs and manifest a reduced expression of inhibitory receptors such as PD-1, CTLA-4 and LAG-3, rendering them relatively impervious to a range of negative regulatory mechanisms. In addition, they exhibit a subtle cytokine expression potential and yet have a great capacity for persistence, expansion and differentiation. Boosting agents such as peptides, if delivered to achieve optimal exposure and TCR-dependent stimulation, can then rapidly drive the expansion and differentiation of DNA-primed CD8^+ ^T cells to peripheral memory/effector cells (CD62L^neg^) that are no longer confined to the lymphatic system and are able to survey peripheral organs. These differentiated cells, nevertheless, simultaneously acquire expression of inhibitory receptors such as PD-1 and are therefore far more susceptible to negative regulatory mechanisms *in vivo*. While boosting would effectively result in activated cells endowed with potent effector capabilities yet prone to exhaustion due to high PD-1 expression, iterative priming would lead to a continuous replenishment of central memory T cells with a low PD-1 expression level and potentiate a renewed source of effector cells upon subsequent boosting. It is also quite possible that co-administration of TLR-ligands with boosting peptide would limit the acquisition and expression levels of PD-1 on effector T cells, thus resulting in a prolonged cellular life-span and enhanced function. This model attempts to explain the synergy between priming and boosting vectors at a single epitope level and the dynamic interplay between various pivotal populations of antigen-specific T cells (such as central and peripheral memory, PD-1^low ^and PD-1^high^) that determines the overall immunity against the intended target (Figure 4B). Furthermore, it provides a rationale for why a precise sequence of administration of different vectors for priming or boosting the immune response is a crucial pre-requisite for an enhanced specific T cell response, measured systemically (Figure 4B) or within lymphoid organs (Figure 5).

The finding that the low PD-1 expression profile afforded by DNA vaccination could be reproduced by intra-lymph node immunization with limited amounts of peptide and TLR stimulation sheds light on the mechanism of action of DNA vaccines and their potency as priming agents in terms of: i) the importance of extended yet reduced levels of antigen exposure; and ii) a role for TCR-independent stimulation through TLRs. However, it should be noted that within this model (Figure 4 and 5) DNA vaccines alone have a limited capability to elicit robust immune responses in homologous prime-boost regimens, as supported by experimental clinical observations as well as mechanistic studies [15-17]. Instead, we argue that the use of DNA vaccines for the purpose of priming high quality antigen-specific CD8^+ ^T cell responses is a viable and highly promising strategy. For example, one could envisage alternating the administration of a DNA vaccine with other vectors such as peptides, recombinant proteins, or viruses for the purpose of inducing and periodically replenishing low PD-1-expressing central-memory T cells and then, through boosting, maintaining a pool of highly functional effector cells. Thus, such heterologous prime-boost regimens would ensure the presence of desirable T cell populations over a longer interval, prevent overall immune exhaustion, and maximize the clinical effect in a therapeutic setting such as cancer, where endogenous antigen exposure alone may not be sufficient to initiate or maintain a clinically relevant immune response.

**Figure 6:**
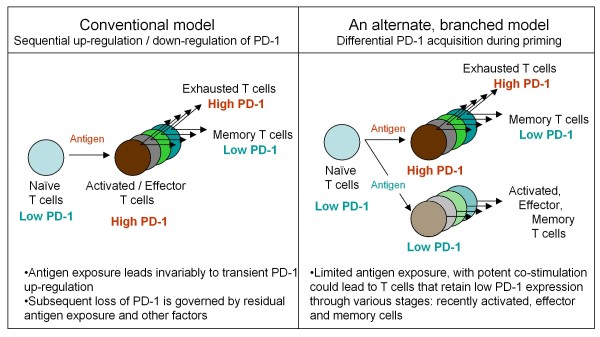
**Another dimension to the immune regulation of CD8^+ ^T cells based on PD-1 expression**. The lack of PD-1 up-regulation during priming may define a separate differentiation lineage. A current model (left side) depicts activation and differentiation of T cells, in relation to PD-1 expression, as a sequential upregulation and downregulation of PD-1, respectively. In this model, activated T cells unavoidably go through a stage in which they are sensitive to PD-1/PD-1L dependent negative regulatory mechanisms. Conversely, in the model depicted on the right side, the acquisition of PD-1 during T cell priming could be limited - depending on the priming regimen - thus yielding T cells that are not as susceptible to negative regulatory mechanisms associated with continuous or repeated antigen exposure. Thus, based on this model - and supported by recent evidence (42, 48) - immediate boosting would yield substantially higher immunity as opposed to immune 'exhaustion'. This enables the development of shortened immunization regimens utilizing a heterologous prime-boost strategy.

There may be a more fundamental aspect to these findings related to the basic immune regulatory processes of CD8^+ ^T cell response in general. The conventional paradigm has been that, upon antigen priming or stimulation, responding T cells go through an unavoidable phase during which they upregulate PD-1 [53]. During the next phase when the antigen exposure subsides, a minor subset of T cells down-regulate PD-1 and become memory cells, while the larger pool of effector cells extinguishes through a range of mechanisms leading to cellular apoptosis. Conversely, if the antigen exposure persists or elevates beyond a certain threshold, the specific T cells would undergo 'exhaustion' mediated primarily by PD-1, a quite distinctive mechanism of immune regulation [54,55]. In the specific case of HIV, PD-1-induced interleukin-10 production by monocytes impairs CD4^+ ^T cell activation, further amplifying the pathogenesis [55]. Instead of supporting this 'serial' differentiation model (with sequential up-regulation and down-regulation of PD-1), our results support a 'branched' differentiation model for CD8^+ ^T cells [56,57]. Accordingly, certain immunization regimens or immune threats expose lymphatic organs to continuously low levels of antigen and robust co-stimulation signals, which result in T cells that fail to up-regulate PD-1 or other co-inhibitory molecules, are less susceptible to negative regulatory mechanisms, and instead are in a prolonged state of 'readiness' (Figure 6). We can only speculate that this mechanism of immune regulation, based on a separate PD-1^low ^T cell branch, evolved to provide the immune system with an advantage over highly virulent microbes that easily penetrate the outer layers of innate immune defense.

## Optimization of prime-boost vaccines based on PD-1 expression and functional avidity of T cells

The body of evidence discussed in this review supports three major conclusions. First, a heterologous prime-boost vaccine should ideally encompass a priming regimen that results in the induction of specific T cells co-expressing low levels of inhibitory receptors. Thus, following a heterologous boost (even within a short time-frame), these cells would expand and differentiate into effector cells rather than being subjected to negative regulatory mechanisms. Secondly, emerging data suggests that DNA vaccines have the capability to elicit low PD-1 expressing CD8^+ ^T cells of central-memory phenotype, a process reproduced by repeat intra-lymph node exposure to minute levels of antigen in the presence of robust TLR9 stimulation. Third, this evidence points to a new dimension of immune homeostasis determined by a tight and synchronized control of inhibitory molecule expression by CD8^+ ^T cells during antigen exposure. This facet of immune homeostasis would shape - as a function of antigen exposure and co-stimulation - the delicate balance between long-lived, readily expandable CD8^+ ^T cells and short-lived T cells that are subject to exhaustion or other negative regulatory mechanisms, in a manner fitting the immunological threat.

Key prerequisites for an effective immune response-to control disseminated tumors for example-are not only the sheer numbers of tumor-associated antigen (TAA)-specific T cells but their quality or capability to recognize and eradicate cancerous cells. The latter depends on the functional avidity of the T cells [58] as well as their polyfunctionality [59] in an environment plagued by immune evasion mechanisms [60]. An interesting fact is that the induction of high magnitude immunity, generally requiring exposure to significant antigen doses, may result in a lower proportion of high avidity T cells [61,62]. This is quite important since tumor cells as well as chronically infected cells may display significantly reduced amounts of antigen which are 'invisible' to vaccine-specific T cells displaying low functional avidity, yet readily quantifiable with current immune monitoring techniques [63].

**Figure 7:**
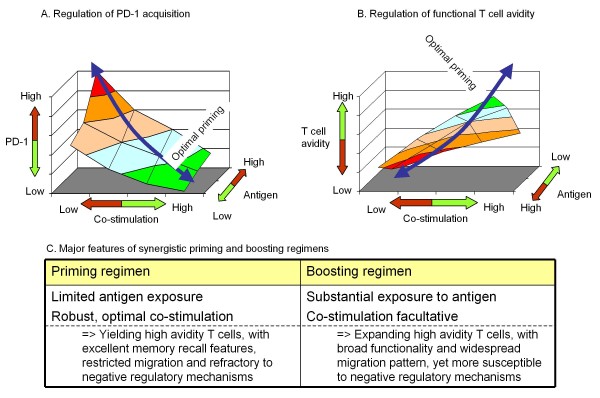
**Co-regulation of PD-1 acquisition and functional avidity of T cells during immune priming**. A and B show schematically the key parameters controlling two complementary features of T cells resulting from immune priming: PD-1 expression (A) and the functional avidity (B). Effective priming warrants optimal, balanced exposure to TCR-dependent and independent stimuli ("green zone") resulting in T cells with a desired effector profile upon boosting. Please note the inverse relationship between functional avidity and the amount of antigen. The table (bottom) depicts the major, synergistic features of priming and boosting vectors/regimens, as a pre-requisite to designing superior vaccination strategies. The model is based on published research (eg. refs [40,42,48,59,60]).

The interplay between antigen exposure and co-stimulation, with relevance to the acquisition of PD-1 and preferential induction of high avidity T cells, is represented in Figure 7. Altogether, this model lays out a novel paradigm for designing heterologous prime-boost vaccines and potentially optimizing homologous prime-boost regimens, applicable to difficult and unmet indications such as cancer and chronic viral infections. The core principle of this paradigm is the selection and optimization of the priming vector or regimen, to achieve induction of specific T cells that meet the following three criteria:

1) have low expression of co-inhibitory receptors (PD-1);

2) display a central memory phenotype;

3) have a high TCR functional avidity.

This new paradigm assumes that the selection of vectors is such that it would not result in a deleterious anti-vector immunity. The priming strategy could then be matched with heterologous vectors that expand and/or differentiate the primed cells to therapeutically useful effector T cells or, alternatively, with homologous boosting leading to much higher antigen exposure than during priming. Notably, the latter, which could be a less expensive strategy since it relies only on one vector, is supported by the observation that exposure to gradually higher levels of antigen (starting from minute amounts) over a fairly short interval of just a few days achieved an unexpectedly robust immune response [64], usually only attainable by live virus infection or heterologous prime-boost vaccination. A similar principle could be applied to homologous prime-boost regimens encompassing naked DNA as primer followed by electroporated DNA as a boosting agent [65]. Effective priming may also be achievable through intradermal delivery of DNA as shown in a model of human skin tattooing [66].

In light of the scarcity of antigen-specific immune interventions that achieve clear-cut therapeutic benefits in cancer and chronic infections, there is clearly a need for advanced vaccine approaches that undergo rigorous testing and afford objective, quantifiable clinical responses. The paradigm outlined in this review shifts the focus from the overarching objective of inducing high numbers of vaccine-specific lymphocytes to that of generating highly efficacious T cells that are potent in adverse environments brought about by continuous antigen exposure or non-antigen related immune inhibitory mechanisms. Furthermore, these observations warrant a revision of current immune monitoring approaches in an effort to more accurately measure, predict and optimize the efficacy of active immunotherapies.

## Conclusions

Mounting evidence supports a different model defining the mechanisms of heterologous prime-boost immunization at the epitope level. In summary, effective priming necessitates low PD-1-expressing central memory T cells and boosting results in their expansion and conversion to effector T cells equipped with broad migratory and functional capabilities. This mechanism is most likely linked to a new dimension of immune homeostasis with a possible role in ensuring the 'response-readiness' of CD8^+ ^T cells, depending on the nature and magnitude of the immunological threat. Finally, this paradigm suggests a series of valuable criteria to guide the design of new immunization regimens.

## Competing interests

AB, ZQ, RW and MO are full time employee receiving salaries from MannKind Corporation. KAS is a paid consultant of MannKind Corporation.

## Authors' contributions

AB wrote the first draft. ZQ, RW, MO, and KAS provided comments and edits for revisions. All authors agreed on the final manuscript.
